# The Role of Blood Flow in Corpus Luteum Measured by Transvaginal Two-Dimensional and Three-Dimensional Ultrasound in the Prediction of Early Intrauterine Pregnancy Outcomes

**DOI:** 10.3389/fphar.2019.00767

**Published:** 2019-07-09

**Authors:** Huijuan Han, Xinhai Mo, Yuqin Ma, Yuqing Zhou, Bo Zhang

**Affiliations:** ^1^Department of Ultrasound, Shanghai Changning Maternity and Infant Health Hospital, Shanghai, China; ^2^Department of Ultrasound in Medicine, Shanghai East Hospital, Tongji University School of Medicine, Shanghai, China

**Keywords:** transvaginal two-dimensional ultrasound, transvaginal three-dimensional ultrasound, receiver operator characteristic curve, early intrauterine pregnancy, corpus luteum

## Abstract

**Objective:** The purpose of this study was to explore the application of transvaginal two-dimensional and three-dimensional power Doppler ultrasound in pregnancy corpus luteum to predict the final outcome of early intrauterine pregnancy.

**Methods:** This is a prospective observational cohort study. Six hundred early intrauterine pregnant women in Shanghai Changning Maternity and Infant Health Hospital were selected as the research objects from January 2015 to December 2015. According to the follow-up of 12 weeks, these pregnant women were divided into the normal pregnancy group (group A, *n* = 512) and the terminational pregnancy group (group B, *n* = 88). They all underwent both transvaginal two-dimensional ultrasound and three-dimensional power Doppler ultrasound to obtain relevant parameters of corpus luteum, namely, the average diameter of the corpus luteum (D), resistance index (RI), pulsatility index (PI), corpus luteum volume (V), vascularization index (VI), blood flow index (FI), and vascularized blood flow index (VFI). Among them, V, VI, FI, and VFI were calculated with the virtual organ computer-aided analysis method. Receiver operator characteristic (ROC) curves were drawn. The corresponding diagnostic cut-off, sensitivity, and specificity were calculated and compared.

**Results:** Compared with group A, the D, V, VI, FI, and VFI of corpus luteum in group B were statistically significant lower while RI and PI were statistically significant higher (*P* < 0.05). The diagnostic cut-off values in the prediction of early intrauterine pregnancy outcomes were D: 14.48, RI: 0.56, PI: 0.81, V: 3.89, VI: 21.48, FI: 38.99, and VFI: 10.21 respectively, and the sensitivity and specificity were D (99.2%, 67.0%), RI (98.9%, 65.0%), PI (78.4%, 89.1%), V (95.1%, 78.4%), VI (74.%, 90.9%), FI (91.8%, 90.9%), and VFI (93.9%, 87.5%) respectively. The area under the ROC curve of the combined index (RI + FI) was 0.963, which was not significantly higher compared with any single index, and both the sensitivity and specificity were 94.3%.

**Conclusion:** Both transvaginal two-dimensional and three-dimensional ultrasonography are of high diagnostic value in predicting the early intrauterine pregnancy outcomes.

## Introduction

Early spontaneous abortion is one of the common complications during pregnancy, which may be attributed to embryonic factors, maternal factors, immune dysfunction, and environmental factors (Hassold et al., 2012). The corpus luteum (CL) is the primary organ producing progesterone during early first trimester, after which the placenta is capable of producing enough progesterone (Niswender et al., 2000). Continuing rescue of the CL by human chorionic gonadotropin (hCG) is essential, otherwise declining progesterone secretion is detrimental to maintaining pregnancy (Jarvela et al., 2008). Therefore, the normal function of CL is vital for pregnancy. However, its function may be affected by the abnormal oosperm, embryo implantation dysfunction, or other systemic disease (Wathes et al., 2003). Based on the researches at home and abroad, the capacity of the CL to produce progesterone is highly related to the extent of its vascular network (Tamanini and De, 2010). That is to say the more blood flow exists in CL, the more progesterone can be produced. The amount of vasculature accounts for more than 20% of the total volume of the CL, exceeding that of any other tissue, which enables to obtain oxygen, nutrients, and hormone precursors necessary to synthesize and release large amounts of progesterone (Henriquez et al., 2016). Therefore, it is crucial to detect the functional status of the CL in clinical work. Clinical symptoms of vaginal bleeding or abdominal pain and low level of hCG or progesterone are usually considered as a sign of the CL insufficiency (Wang et al., 2015). But the above performance is lack of specificity and accuracy to assist diagnosis and therapy. Hence, some accurate, objective, convenient and non-invasive parameters of the CL function are currently needed for early prediction of pregnancy outcome. It is commonly recognized that transvaginal two-dimensional ultrasound with color Doppler flow imaging (CDFI) is the optimum non-invasive imaging method for evaluating the sonographic features and vascularization of CL (Durfee and Frates, 2015). Doppler flow study with its indices such as pulsatility index (PI) and resistance index (RI) provides important information about perfusion and angiogenesis in the ovarian follicles (Guiot et al., 2008). Transvaginal three-dimensional ultrasound with power Doppler is a new emerging method of studying vascularization recently (Jokubkiene et al., 2012). It might better reflect vascular changes than two-dimensional color or power Doppler, because vascular changes in a whole organ can be assessed through blood flow velocity in one or a few vessels, and the results of quantitative analysis can be acquired by using the virtual organ computer-aid analysis (VOCAL) software (Jarvela et al., 2003). The histogram facility of the Vocal software automatically obtains three vascularity indices, namely vascularization index (VI), flow index (FI), and vascularization flow index (VFI), which potentially can reflect the vascular density, blood flow, and tissue perfusion respectively. So the quantification of complete blood flow of the region of interest from the analysis of power Doppler signals can be fully studied (Martins et al., 2011). To the best of our knowledge, there are few published studies where transvaginal two-dimensional and three-dimensional ultrasound have been used simultaneously to study the CL vascularity and its relationship with the pregnancy outcome in early first trimester. The aim of this study was to explore the prognostic value of transvaginal two-dimensional and three-dimensional ultrasound in predicting early intrauterine pregnancy outcome by measuring the blood flowing parameters related to the CL.

## Materials and Methods

### Subjects

This is a prospective observational cohort study. Six hundred patients with early pregnancy and fertility requirements from January 2015 to December 2015 in Shanghai Changning Maternity and Infant Health Hospital were selected as the research objects with average age (27.1 ± 10.5) years old and average menstrual time (47.61 ± 6.18) days. Inclusion criteria: 1) Plain menstrual rules; 2) Exclusion of gynecological diseases such as uterine fibroids, adenomyosis, and ovarian cysts; 3) Exclusion of congenital uterine malformations. 4) Exclusion of repeated pregnancy loss, adverse pregnancy outcome, polycystic ovary syndrome (PCOS), and other systemic disease. According to the follow-up of 12 weeks, these pregnant women were divided into the normal pregnancy group (group A, *n* = 512) and the terminational pregnancy group (group B, *n* = 88). Informed consent was obtained from all women after a full explanation of the objectives of the study. The research protocol was approved by the Ethics Committee of Shanghai Changning Maternity and Infant Health Hospital (protocol number: CNFBLLYR-20150102).

### Equipment and Sonographer

All data were acquired using a US GE Volusion E8 ultrasound system equipped with a 4–9 MHz transvaginal transducer. The Vocal software was provided for automatically measurement of relevant vascular parameters. Identical fixed pre-installed power Doppler ultrasound settings were used in all selected women: 1) High quality imaging (Qual high); 2) Frequency (Frq mid); 3) Color gain without overflow (gain 0); 4) Pulse repetition frequency (PRF) 0.3 kHz; 5) Mixing ratio (mix 20%/80%); 6) The wall motion filter to ‘‘low 1’’. All ultrasound examinations were performed by the same dedicated sonographers with more than 5 years of working experience.

### Ultrasound Examination

**Figure 1:**
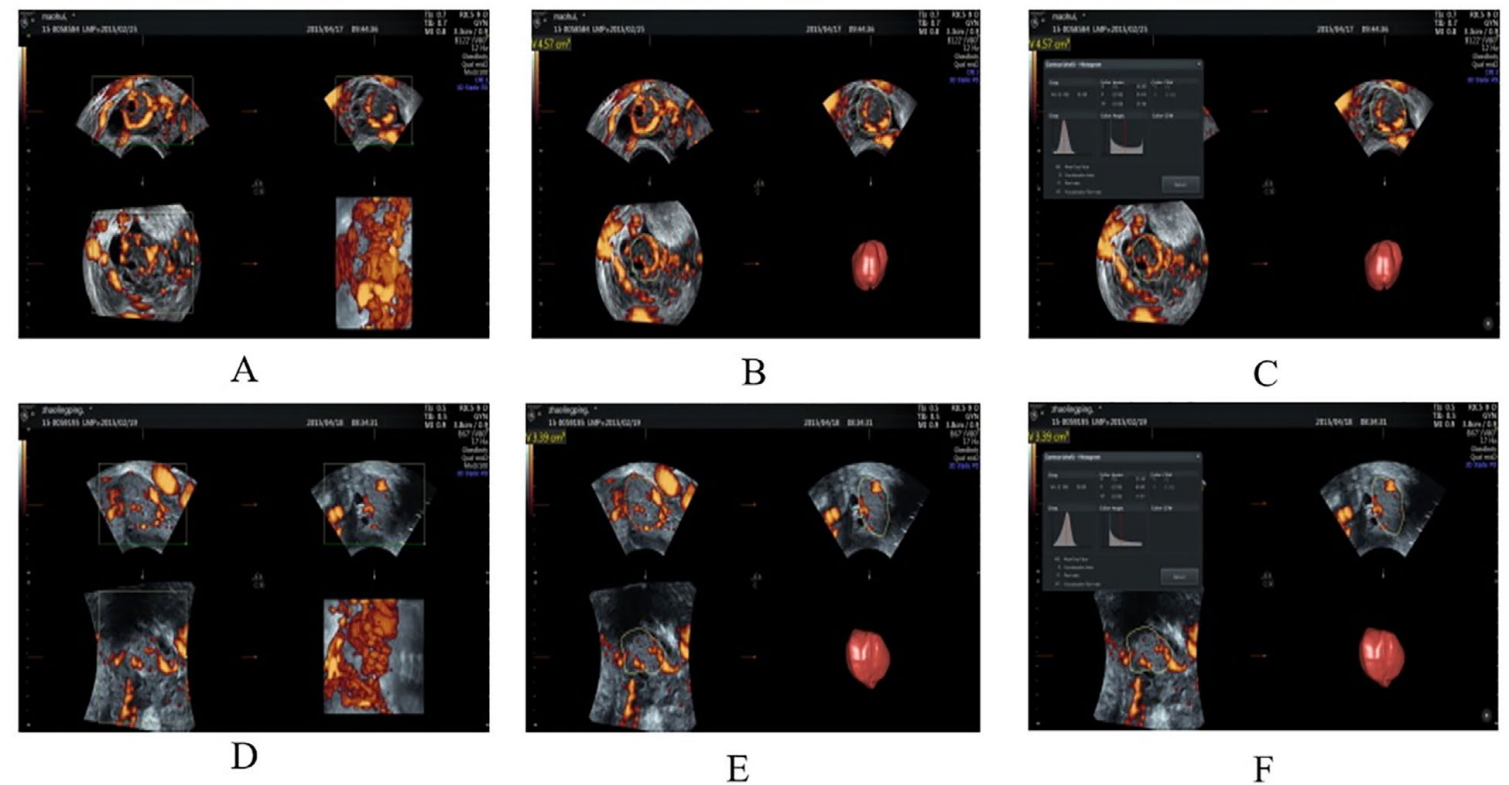
Three-dimensional ultrasound images of corpus luteum in group A and group B. **(A, B)** The 3D-PD ultrasound images of the corpus luteum in group A (27 y, G1P0, menolipsis 69 days, no symptoms of abdominal pain or vaginal bleeding, intrauterine pregnancy, having yolk sac, germ, and fetal heart beating). **(C)** The histogram of gestational corpus luteum in the same patient. **(D, E)** The 3D-PD ultrasound images of the corpus luteum in group B (34 y, G1P0, menolipsis 59 days, with the symptoms of abdominal pain and vaginal bleeding, intrauterine pregnancy, having yolk sac and germ, no fetal heart beating). **(F)** The histogram of gestational corpus luteum in the same patient. 3D-PD indicates the three-dimensional power Doppler.

All pregnant women were examined in the lithotomy position with an empty bladder. The ultrasound probe was introduced slowly into the vagina, and care was taken to avoid exerting undue pressure. First, in the grayscale mode, routine transvaginal two-dimensional ultrasound was first conducted to identify normal intrauterine pregnancy and seek for the pregnancy CL, scan, and record the average diameter of the corpus luteum (D). Next, observe the blood flow signal of CL with CDFI, adjust the color scale to the best mode, and place the sampling line at the most vivid color of the blood flow signal to start. Pulse Doppler detection measures the resistance index (RI) and the pulsatility index (PI) after acquiring more than three consecutive spectra. Then, based on the satisfactory view, the 3D ultrasound mode was switched on, then adjust the position and size of the sampling frame and start the VOCAL program, for A, B, and C as shown in **Figure 1**. The A plane (vertical axis) in the plane was manually outlined, and the rotation angle of the adjacent section was set to 30°. Once six contours had been drawn, the volume of the CL (V) was calculated automatically. Using the histogram facility of Vocal software, three vascular indices were generated: vascularization index (VI) means the proportion of the volume showing a flow signal in the fetal brain; blood flow index (FI) is the average flow signal intensity inside the fetal brain and vascularized blood flow index (VFI) is a combination of the information concerning vessel presence and the amount of flow obtained by multiplying VI and FI.

### Statistical Analysis

All WSS data were analyzed by Microsoft Excel and SPSS 22.0 software (SPSS Inc., Chicago, IL, USA). Shapiro–Wilk test was used to assess the distribution of continuous variables. Results were expressed as frequencies and percentages for categorical variables, mean ± standard deviation (SD) for normally distributed variables, and median (interquartile range) for non-normally distributed variables. Continuous variables were compared by one-way analysis of variance with Bonferroni *post hoc* test or Kruskal–Wallis tests. Mann–Whitney *U* test for nonparametric variables. Receiver operating characteristic (ROC) curves was used to evaluate the individual ability of the parameters to predict the pregnant outcomes. Areas under the curve (AUC) for each ROC curve were calculated and compared by *Z* test. *P* < 0.05 difference was statistically significant.

## Results

Overall, 600 pregnant women were detected in our study. The 512 pregnant women were enrolled in group A and the median (interquartile range) age was 29(5) years in group A, whereas in group B of 88 pregnant women, the median age was 29(6) years. There was no significant difference (*P* < 0.05) when comparing group A with group B.

### Transvaginal Two-Dimensional Parameters of Corpus Luteum

The transvaginal two-dimensional parameters of the two groups were compared in **Table 1**. The mean D value was significantly higher in group A (21.06 ± 3.08 mm), compared to the value in group B (13.92 ± 2.86 mm; *P* < 0.05). While the RI and PI were significantly lower in group A (0.54 ± 0.22 and 0.72 ± 0.21, respectively) than those values of group B (0.61 ± 0.32 and 0.86 ± 0.14, respectively; *P* < 0.05).

**Table 1:** Transvaginal two-dimensional parameters of corpus luteum in two groups.

Variable	Group A	Group B	*P* value
D (mm)	21.06 ± 3.08	13.92 ± 2.86	0.000*
RI	0.54 ± 0.22	0.61 ± 0.32	0.000*
PI	0.72 ± 0.21	0.86 ± 0.14	0.000*

*means P < 0.05; D indicates the average diameter of the corpus luteum; RI, the resistance index; and PI, the pulsatility index.

### Transvaginal Three-Dimensional Parameters of Corpus Luteum

The transvaginal three-dimensional parameters of the two groups were compared in **Table 2**. The V, VI, FI, and VFI were significantly higher in group A (6.64 ± 1.98 mm^3^, 26.16 ± 11.51, 49.58 ± 21.71 and 14.60 ± 3.62, respectively), compared to group B (3.58 ± 1.58 mm^3^, 15.61 ± 6.55, 31.268 ± 6.31 and 6.92 ± 3.12, respectively; *P* < 0.05).

**Table 2:** Transvaginal three-dimensional parameters of corpus luteum in two groups.

Variable	Group A	Group B	*P* value
V (mm^3^)	6.64 ± 1.98	3.58 ± 1.58	0.000*
VI	26.16 ± 11.51	15.61 ± 6.55	0.000*
FI	49.58 ± 21.78	31.26 ± 6.31	0.000*
VFI	14.60 ± 3.62	6.92 ± 3.12	0.000*

*means P < 0.05; V indicates corpus luteum volume; VI, vascularization index; FI, blood flow index; VFI, vascularized blood flow index.

### ROC Curves for Both Transvaginal Two-Dimensional and Transvaginal Three-Dimensional Parameters

**Figure 2:**
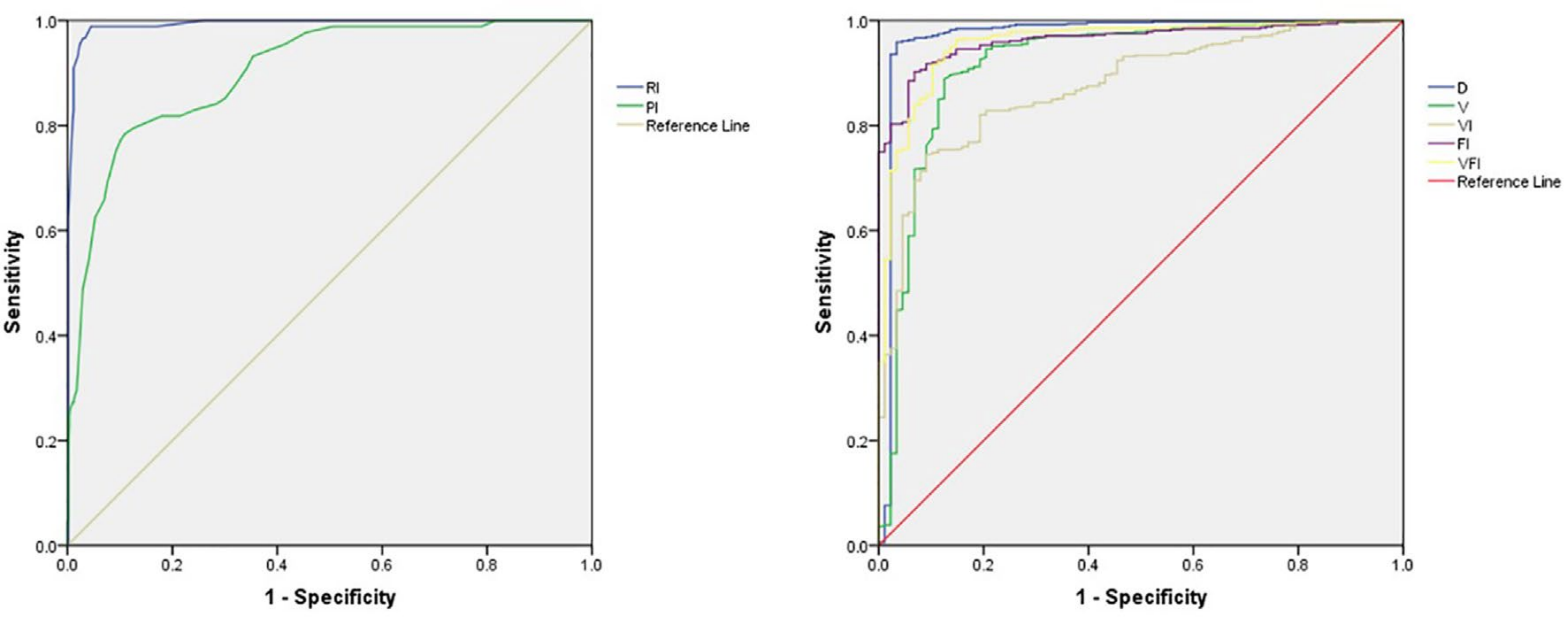
Receiver operator characteristic (ROC) curves for RI, PI, D, V, VI, FI, and VFI. RI indicates the resistance index; PI, the pulsatility index; D, the average diameter of the corpus luteum; V, corpus luteum volume; VI, Vascularization index; FI, blood flow index; and VFI, vascularized blood flow index.

As shown in **Figure 2**, ROC curves were drawn according to the parameters of group A and group B. The diagnostic cut-off value, sensitivity, and specificity of all parameters were described in **Table 3**.

**Table 3:** AUC for ROC curves for D, RI, PI, V, VI, FI, and VFI.

Variable	AUC	95% CI
D (mm)	0.970	0.940–1.000
RI	0.992	0.985–0.999
PI	0.903	0.870–0.936
V (mm^3^)	0.917	0.877–0.985
VI	0.875	0.840–0.910
FI	0.962	0.948–0.977
VFI	0.957	0.935–0.979

AUC indicates areas under the curve; ROC, receiver operator characteristic; CI, confidence interval; D, the average diameter of the corpus luteum; RI, the resistance index; PI, the pulsatility index; V, corpus luteum volume; VI, vascularization index; FI, blood flow index; and VFI, vascularized blood flow index.

### Binary Logistic Regression Analysis

**Figure 3:**
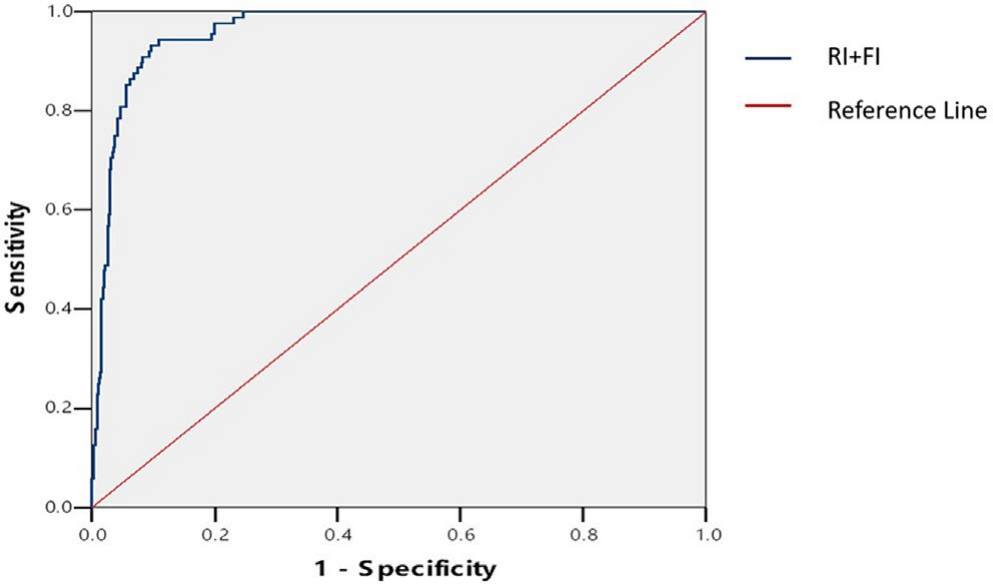
ROC curve for joint parameter (RI+FI). RI indicates the resistance index; and FI, blood flow index.

From the above analysis, we found that the AUC for ROC curve of RI among the two-dimensional parameters and FI among three-dimensional parameters were significantly higher. The ROC curve analysis of the joint indicator of RI and FI was performed by the binary logistic regression analysis as shown in **Figure 3**. The joint indicator of RI and FI was not superior to individual RI or FI in the prediction of early intrauterine pregnancy outcomes, with an AUC not significantly higher (**Table 4**).

**Table 4:** AUC for ROC curve for RI, FI, and RI+FI.

Variable	AUC	95% CI	*P* value
RI	0.992	0.985−0.999	<0.05*
FI	0.962	0.948−0.977	<0.05^†^
RI+FI	0.963	0.948−0.978	−

*P < 0.05 versus RI+FI; ^†^P > 0.05 versus RI+FI; AUC indicates areas under the curve; CI, confidence interval; RI, the resistance index; and FI, blood flow index.

## Discussion

Spontaneous abortions are serious life events for both family and society. The frequency of early spontaneous abortions is estimated to be 10–15% of clinically recognized pregnancies and as many as 30% of clinically unrecognized pregnancies (Baba et al., 2011). Although chromosomal abnormalities of the fetus or increasing maternal age are the major risk factors of early spontaneous abortions, it is not uncommon to attribute such adverse event to the CL insufficiency in clinic (Duru and Nagadeepti, 2013). At present, the prognosis of intrauterine pregnancy mostly depends on the clinical symptoms such as vaginal bleeding and abdominal pain and low level of hCG or progesterone, which have poor specificity and sensitivity (Dinelli et al., 2014). It is well-known that the CL continues to grow under the stimulation of hCG after fertilization and is the only source to produce progesterone to maintain pregnancy before the formation of placenta (Sgs et al., 2017). An abnormal endometrial lymphocyte pattern occurs in infertile women affected by PCOS, together with profound impairment of the endometrial cytokine balance, even after normal ovulation (Matteo et al., 2010). The cut-off value for serum progesterone (35 nmol/L) demonstrated clinical relevance and allow clinicians to stratify patients into high and low risk groups for spontaneous miscarriage (Lek et al., 2017). Therefore, the normal function of CL is vital for pregnancy. In early pregnancy, the ovary can be induced to sprout new blood vessels which emitting collateral circulation into the CL, so the blood perfusion around the CL is significantly abundant (Tamura et al., 2008).

Transvaginal sonography has unique advantages in detecting the CL function by measuring its morphology, echo, size, volume, as well as evaluating the vascularity with color Doppler velocimetry. Previous researches did not reach an agreement about the morphology and echo of CL, which may attribute to individual difference (Pareja et al., 2010; Durfee and Frates, 2015). Therefore, we did not research the morphology and echo of CL in our study. Three-dimensional ultrasound scanning and power Doppler angiography have been introduced in clinical practice recently. This technique overcomes some limitations of conventional B-mode and color/power Doppler ultrasound scanning. It was relatively independent with high sensitivity and reproducibility, which can detect small and slow blood vessels in three-dimensional space of the CL (Martins, 2010). With the three-dimensional power Doppler ultrasound, ultrasound parameters VI, VFI, and FI can be measured and statistically analyzed in the histogram facility of the Vocal software so as to reduce the error caused by subjective judgment (Pellaers et al., 2017).

The major findings of our study were: a) the RI and PI were significantly lower in normal pregnancy group, while the D, V, VI, FI, and VFI were significantly higher; b) the AUC for ROC curve of RI among the two-dimensional parameters and FI among three-dimensional parameters were significantly higher; c) the joint indicator of RI and FI was not superior to individual RI or FI in the prediction of early intrauterine pregnancy outcomes. Our results were in accordance with the previous study (Pareja et al., 2010), when comparing the diameter and volume of the CL of normal pregnant women with those who aborted, found lower diameter and volume in cases of abortion. Many studies had demonstrated that RI and PI were significantly higher in patients of abortion (Applebaum, 1995; Durfee and Frates, 2015). RI is an indicator of blood circulation resistance. The smaller the RI value, the more abundant the blood flow. In the study by Zhang et al. (2016), VI, FI, and VFI were significantly higher in normal pregnancy group, which was consistent with our study. On contrary, Ahmad et al. (2015) found there was no significant difference by comparing the VI between those who aborted and those with good outcome. As the reflection of the degree of vascular blood supply, the higher VI, FI, and VFI are, the richer newborn blood vessels are within the CL (Alcãzar, 2008). This is consistent with our findings. The joint indicator of RI and FI was not superior in the prediction of early intrauterine pregnancy outcomes, with an AUC not significantly higher. It is probably because either RI or FI has higher specificity and sensitivity. From the results of the study, among all the parameters, the RI and FI can better reflect the blood perfusion of the CL and have a high guiding value for prognosis estimation of pregnant outcomes. The findings in our study may be helpful for obstetricians and gynecologists in early diagnosis, timely prevention, and effective therapy of spontaneous abortions.

There are certain limitations in our study. Firstly, the fetus of group B did not undergo karyotype analysis, although the incidence of aneuploid pregnancy is relatively low. Secondly, it is prone to bring errors in the identification of the contour edge of CL by manual tracing which will affect the software’s calculation of the volume and blood perfusion. Therefore, we should improve the above operations as much as possible to get more accurate statistical results in the future research.

## Conclusion

Both transvaginal two-dimensional and three-dimensional ultrasonography are of high diagnostic value in predicting the early intrauterine pregnancy outcomes. Therefore, they provide a useful tool for early and accurate diagnosis and guiding for treatment timely in clinic.

## Ethics Statement

This study was carried out in accordance with the recommendations of World Health Organization guidelines with written informed consent from all subjects. All subjects gave written informed consent in accordance with the Declaration of Helsinki. The protocol was approved by the Ethical Committee of Shanghai Changning Maternity and Infant Health Hospital.

## Author Contributions

HH and XM performed the literature review. HH and YZ carried out echocardiography measurements. YZ and BZ checked the validity of data. HH and YM analyzed the data. BZ supported the experiments financially. HH, XM and YM wrote the manuscript. XM and YM revised the manuscript. All authors read and approved the final manuscript.

## Funding

The study was funded by the Pudong Health Bureau of Shanghai (Grant No. PWZbr2017-09), and the National Natural Science Foundation of China (Grant No. 81401428, 81571693, and 81871361).

## Conflict of Interest Statement

The authors declare that the research was conducted in the absence of any commercial or financial relationships that could be construed as a potential conflict of interest.
